# Root-like enamel pearl: a case report

**DOI:** 10.1186/1752-1947-8-248

**Published:** 2014-07-09

**Authors:** Xiao-quan Mao

**Affiliations:** 1 Department of Endodontics, Stomatological Center, Affiliate Haikou Hospital, Xiangya Medical School, Central South University, Haikou 570208, Hainan, P.R. China

**Keywords:** Cone beam computed tomography, Enamel pearl, Periapical radiographic images

## Abstract

**Introduction:**

In general, enamel pearls are found in maxillary molars as a small globule of enamel. However, this case report describes an enamel pearl with a prolate spheroid shape which is 1.8mm wide and 8mm long. The different type of enamel pearl found in my clinic has significantly improved our understanding of enamel pearl etiology and pathophysiology.

**Case presentation:**

A 42-year-old Han Chinese woman with severe toothache received treatment in my Department of Endodontics. She had no significant past medical history. A dental examination revealed extensive distal decay in her left mandibular first molar, tenderness to percussion and palpation of the periradicular zone, and found a deep periodontal pocket on the buccal lateral. Vitality testing was negative. Periapical radiographic images revealed radiolucency around the mesial apex. Cone beam computed tomography detected an opaque enamel pearl in the furcation area with a prolate spheroid shape of 1.8mm wide and 8mm long.

**Conclusion:**

The enamel pearl described in this case report is like a very long dental root. Cone beam computed tomography may be used for evaluating enamel pearls.

## Introduction

Enamel pearls are ectopic deposits of enamel which are located at the furcation area and near the cemento–enamel junction. In general, they are most commonly observed in molars, in particular maxillary molars. They appear as small globules of enamel firmly adherent to the tooth’s root surface and as well-defined radio-opaque nodules. Internal enamel pearls present as well-defined circular areas of radiodensity extending from the enamel–dentin junction to the underlying coronal dentin.

## Case presentation

**Figure 1:**
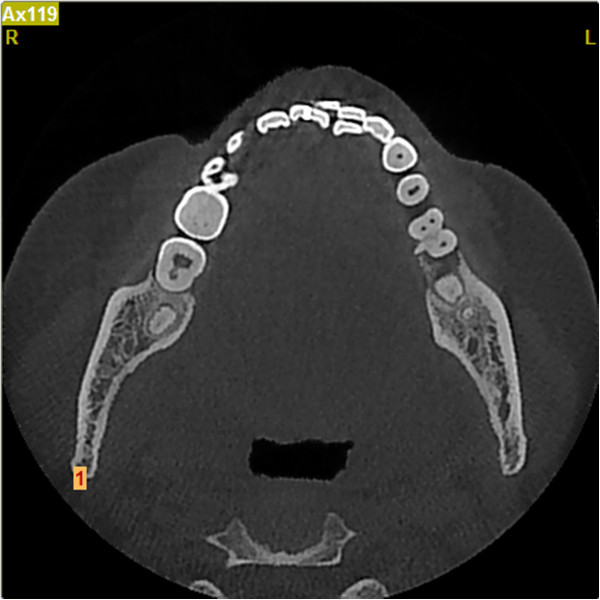
Enamel pearl in furcation area.

**Figure 2:**
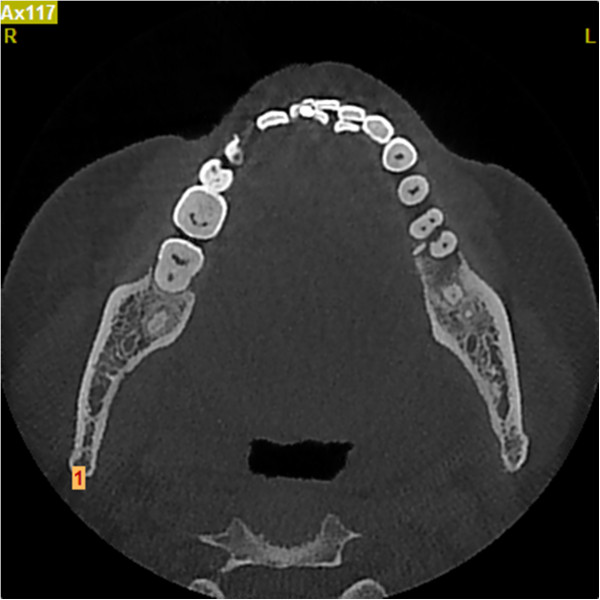
Computed tomography horizontal section view.

**Figure 3:**
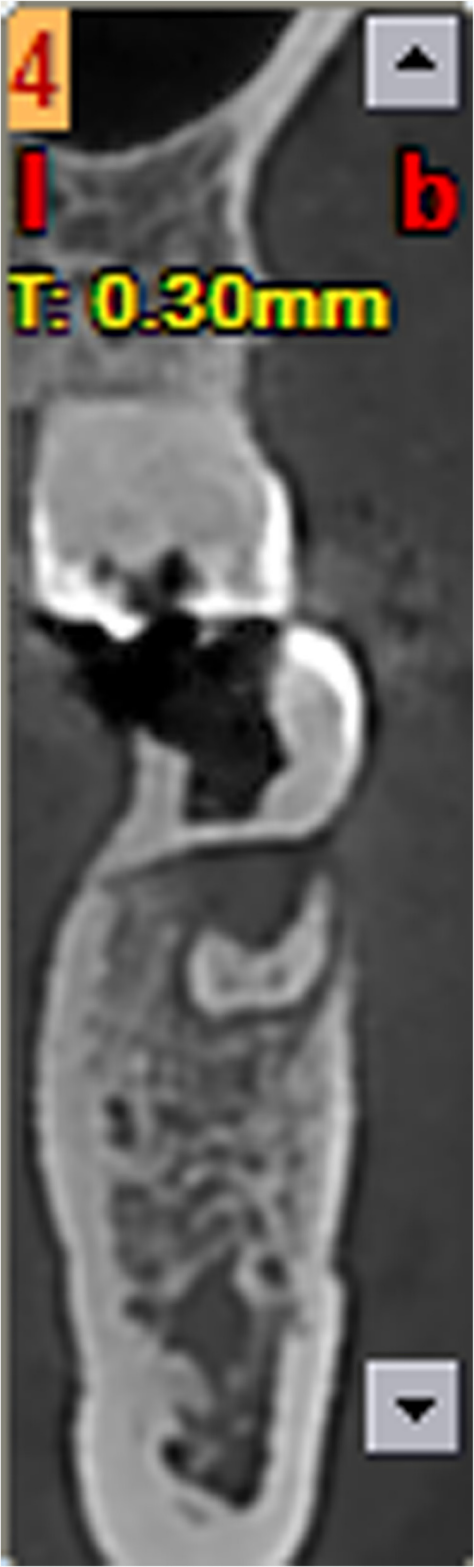
Computed tomography buccolingual section view.

A 42-year-old Han Chinese woman with severe toothache received treatment in my Department of Endodontics. A dental examination revealed extensive distal decay in her left mandibular first molar, tenderness to percussion and palpation of the periradicular zone, and found a deep periodontal pocket on the buccal lateral. Vitality testing was negative. Periapical radiographic images revealed radiolucency around the mesial apex. Cone beam computed tomography (CBCT) detected an opaque enamel pearl in the furcation area with a prolate spheroid shape of 1.8mm wide and 8mm long (Figures 1, 2, and 3).

**Figure 4:**
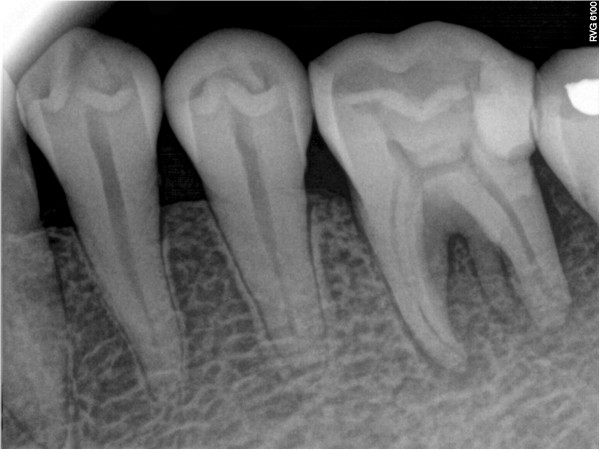
Root-like enamel pearl.

The tooth was diagnosed with symptomatic apical periodontitis with necrotic pulp. After Scandonest® (mepivacaine hydrochloride; Septodont, Inc.) was administered, her tooth was isolated using a rubber dam. As usual, in the initial access cavity, three orifices were found. In addition, a root-like image was found on periapical imaging (Figure 4). Therefore the access cavity was further prepared into a square shape; however, no orifice was found after meticulous exploration of the pulp chamber floor with a hand K-file.

Three canals were instrumented with stainless-steel hand K-files accompanied by X-Smart™ Motor (Dentsply, USA) rotary instrumentation using a crown-down technique. Her root canals were irrigated with sodium hypochlorite 5.25% and dried completely. Camphor phenol was placed as an intracanal dressing. Then, the access cavity was sealed temporarily with zinc oxide.

One week later, she was completely asymptomatic. Her root canals were obturated with gutta-percha (Dentsply, USA) after the working length was radiographically confirmed with gutta-percha.

Subsequently, she was referred to the restorative department for final restoration. Three months later, she had no symptoms.

## Discussion

Enamel pearls are anomalies of enamel on primary and permanent teeth roots that usually appear at furcation areas, especially in maxillary second and third molars [1]. They usually occur as solitary lesions, but two to four enamel pearls have been observed on the same tooth [2]. The prevalence of enamel pearls has been reported to vary between 1.1 and 9.7% [3]. Enamel pearls are not common in teeth with a single root, although there are rare reports of them occurring on the roots of premolars, canines and incisors [4-6]. It is generally accepted that enamel pearls are usually found adherent to the external root surface of the tooth, but on rare occasions they may be detected within the dentin [7]. They vary in size, ranging from 0.3mm to 4mm in diameter [8]. But in this case report, the enamel pearl in our patient was unusually large, 1.8mm wide and 8mm long.

Three types of enamel pearls have been described [9,10]: (1) true enamel pearls, consisting entirely of enamel; (2) composite enamel pearls, or enamel-dentin pearls, containing a core of tubular dentin; and (3) enamel-dentin-pulp pearls, containing a pulp horn, probably extending from the coronal pulp chamber or root canal.

The etiology of enamel pearls remains obscure. The most widely accepted theory is that enamel pearls develop as a result of localized developmental activity of a remnant of Hertwig’s epithelial root sheath which maintains its potential for enamel formation, which could explain how this structure has remained adherent to the root surface during root development [11,12]. It is believed that these cells differentiate into functioning ameloblasts and produce enamel deposits on the root.

CBCT may be helpful to find enamel pearls [13-15]. Enamel pearls are anatomical structures that can lead to clinical implications if associated with the retention of plaque [16] which could prevent periodontal attachment and predispose the area to pocket formation and periodontal disease [17]. In this case, the enamel pearl did not develop tubular dentin and pulp; it is only composed of enamel. Because enamel pearls may contain tubular dentin and/or a pulp chamber, meticulous exploration of the developmental groove in the pulp chamber floor is suggested in order to locate the orifices of canals. Moreover, any dentin projection that could cover an existing orifice should be removed carefully when dental pulp disease is treated.

## Conclusions

The enamel pearl described in this case report is like a very long dental root. In order to ensure the root canal is not missed, the orifice in the pulp chamber floor must be carefully explored. In addition, tubular dentin within enamel pearls should be evaluated using CBCT when these teeth need to be treated.

## Consent

Written informed consent was obtained from the patient for publication of this case report and any accompanying images. A copy of the written consent is available for review by the Editor-in-Chief of this journal.

## Abbreviations

CBCT: Cone beam computed tomography.

## Competing interests

The author declares that he has no competing interests.
